# The effect of gestational diabetes mellitus on pregnancy outcomes in advanced primiparous women: A retrospective study

**DOI:** 10.1097/MD.0000000000037570

**Published:** 2024-03-29

**Authors:** Hong Yang, Chanyun Xiao, Jiahui Tu

**Affiliations:** a Department of Obstetrics, Maternal and Child Health Hospital of Hubei Province, Tongji Medical College, Huazhong University of Science and Technology, Wuhan City, P.R. China.

**Keywords:** Advanced primiparous women, adverse pregnancy outcomes, gestational diabetes mellitus (GDM), pregnancy

## Abstract

Gestational diabetes mellitus (GDM) could have a variable degree of adverse effects on pregnancy outcomes for both pregnant women and newborns. The purpose of the study was to explore the effect of GDM on pregnancy outcomes in advanced primiparous women. A total of 1076 advanced primiparous women were included between January 2020 and December 2022. All these women were divided into the GDM group (*n* = 434) and the non-GDM group (*n* = 642). Variables included baseline characteristics, maternal, and newborn outcomes were collected. The risk of each adverse outcome was analyzed by multivariate logistic regression models. The effect of blood glucose control on pregnancy outcomes was further analyzed among GDM women with good glycaemic control (*n* = 381) and poor glycaemic control (*n* = 53). Analysis of baseline characteristics demonstrated a significant difference in prepregnancy body mass index (median, IQR: 22.27 [20.58–24.44] vs 21.17 [19.53–22.86], *P* < .01) between the GDM group and the non-GDM group. A significantly higher incidence rate of adverse pregnancy outcomes was found in advanced primiparous women with GDM, such as polyhydramniosis, premature birth, low-birth weight, macrosomia, and neonatal intensive care unit admission (all *P* < .05). Compared with the non-GDM group, the risk of polyhydramniosis was nearly twice as high in the GDM group (adjusted odds ratio: 1.94, 95% confidence interval: 1.01–3.72, *P* = .04) after adjusted baseline characteristics. Among the GDM group, the women with poor glycaemic control showed a significantly higher incidence rate of polyhydramnios, hypertensive disorders of pregnancy, cesarean delivery, premature birth, low-birth weight, macrosomia, and neonatal intensive care unit admission was significant than the women with good glycaemic control (all *P* < .05). GDM was an independent risk factor for polyhydramnios in advanced primiparous women. At the same time, good glycaemic control in diabetics advanced primiparous women could reduce adverse pregnancy outcomes.

## 1. Introduction

With the rapid socio-economic development of China, the change in the concept of marriage, and the quest for quality of life, there has been an increasing trend in the number of elderly first-time mothers in China annually. Advanced maternal age (AMA) is commonly defined as maternal age ≥ 35.^[1]^ AMA carries reduced fertility, increased demand for assisted reproductive techniques (ART), and an ascending early miscarriage due to having impacts on chromosomal and genetic disorders. During pregnancy, AMA also increases the probability of pregnancy complications, including gestational diabetes mellitus (GDM), fetal growth restriction, stillbirth related, hypertension, preterm birth, disorders of pregnancy, and so forth.^[2]^ Furthermore, the incidence of GDM in women with AMA has been dramatically increasing.^[3,4]^ GDM is a common complication of pregnancy, defined as defined as abnormal glucose tolerance occurring or first detected during pregnancy and without a history of diabetes.^[5]^ A systematic review and meta-analysis by Ye et al^[6]^ demonstrated that GDM had variable adverse effects on pregnancy outcomes in the near and distant future, including the higher incidence of gestational hypertension, cesarean section rate, macrosomia, postpartum hemorrhage, preterm birth, transferred to neonatal intensive care unit (NICU) admission. Compared with women without GDM, the risk for developing type 2 diabetes mellitus (T2DM) was 7.5 times in women with GDM, and their offspring were more likely to develop childhood obesity and T2DM.^[7]^ A previous study reported that good glycaemic control was of great significance for improving pregnancy complications and perinatal conditions.^[8]^ Though numerous studies have explored the relationship between GDM and pregnancy outcomes, the vast majority of studies were focused on the risk of AMA for adverse pregnancy outcomes in AMA in both primiparous and perinatal mothers,^[2,9,10]^ and few studies were reported in GDM women with advanced primiparous women. Our study aimed to examine the relationship between GDM and pregnancy outcomes in advanced primiparous women.

## 2. Materials and methods

### 2.1. Study design and participants

The current study included 1076 eligible pregnant women from January 2020 to December 2022 in the Maternal and Child Health Hospital of Hubei Province. The inclusion criteria were as follows: primiparous women aged ≥35 years old; pregnant women with no history of prepregnancy diabetes; OGTT performed at 24–28 weeks; venous blood monitored during pregnancy and/or peripheral blood glucose; the data during pregnancy were complete. The exclusion criteria were as follows: the maternal age <35 years old; previous history of delivery, twin or multiple pregnancies. The diagnostic criteria of GDM as determined by 2-hours 75-grams OGTT were as follows: fasting blood glucose level of ≥5.1 mmol/L, 1-hour blood glucose level of ≥10.0 mmol/L, or 2-hours blood glucose level of ≥8.5 mmol/L. These advanced primiparous women were divided into the GDM group (*n* = 434) and the non-GDM group (*n* = 642). Subgroup analysis among GDM women was also conducted by the good or poor glycaemic control during pregnancy. Good glycaemic control was defined as fasting plasma glucose <5.3 mmol/L, 2-hours postload plasma glucose <6.7 mmol/L, and HbA1c <5.5%; otherwise, it was poor considered glycaemic control.^[8]^ The 434 GDM women were further divided into women with good glycaemic control (*n* = 381) and women with poor glycaemic control (*n* = 53). The current study was approved by the Ethics Committee of our hospital (No. 2023IEC080).

### 2.2. Data collection

Baseline characteristics were collected, including maternal age, gravidity, prepregnancy weight, height, prepregnancy body mass index (BMI), gestational age, gestational weight gain (GWG), gravidity, and ART. prepregnancy BMI values were classified according to the WHO cutoff points for Asian adults (Supplementary Table 1, http://links.lww.com/MD/L977).^[11]^ GWG was classified following the 2009 Institute of Medicine guidelines (Supplementary Table 2, http://links.lww.com/MD/L978).^[12]^ The maternal and infant outcomes were collected by the follow-up visit in the hospital, including premature rupture of membranes (PROM), polyhydramnios, hypertensive disorders of pregnancy, hypothyroidism, cesarean delivery, postpartum hemorrhage (≥500 mL for vaginal delivery or ≥1000 mL for cesarean delivery), fetal distress (the symptoms of respiratory and circulatory insufficiency caused by intrauterine fetal hypoxia during labor), low-birth weight (considered to ≤2500 grams), macrosomia (≥4000 grams), preterm birth (<37 weeks of gestation), and NICU admission.

### 2.3. Statistical analysis

Statistical analyses were performed using SPSS version 23.0 (SPSS, Chicago, IL, USA). Continuous data with the nonnormal distribution were presented as median (IQR) and analyzed using the Mann–Whitney *U* test. Categorical data were presented as *n* (%) and analyzed using the Chi-square test. The risk of adverse pregnancy outcomes was analyzed using the odds ratio (OR) and 95% confidence interval (CI) by multivariate logistic regression models. Adjustment was made for baseline characteristics of maternal age, height, weight, prepregnancy BMI, GWG, ART, and gravidity. *P* < .05 was considered statistically significant.

## 3. Results

A total of 1076 eligible advanced primiparous women were included in our study. All these women were divided into the GDM group (*n* = 434) and the non-GDM group (*n* = 642). Analysis of baseline characteristics demonstrated a significant difference in prepregnancy BMI (median, IQR: 22.27 [20.58–24.44] vs 21.17 [19.53–22.86], *P* < .01) between the 2 groups. The gestational age in the GDM group was significantly lower than that in the non-GDM group (median, IQR: 38.6 [38.1–39.2] vs 39.1 [38.2–39.5], *P* < .01). Compared with the non-GDM group, the GWG was much lower (median, IQR: 11.50 [8.0–15.0] vs 14.0 [11.0–17.0], *P* < .01) in the GDM group and the GDM group had a larger proportion of inadequate GWG (42.17% vs 25.39%, *P* < .01). Detailed data was shown in Table 1.

**Table 1:** Comparison of baseline characteristics of advanced primiparous women with/without GDM.

Variables	GDM group (*n* = 434)	Non-GDM group (*n* = 642)	*P* value
Maternal age (y), (median, IQR)	36.00 (35.00–38.00)	36.00 (35.00–38.00)	.14
Height (cm), (median, IQR)	160 (158–164)	160 (158–164)	.30
Weight (kg), (median, IQR)	58 (52–63)	55 (50–60)	<.01
Prepregnancy BMI (kg/m^2^), (median, IQR)	22.27 (20.58–24.44)	21.17 (19.53–22.86)	<.01
GWG (kg)	11.50 (8.0–15.0)	14.0 (11.0–17.0)	<.01
GWG categories (%)			**<**.01
Inadequate	183 (42.17)	163 (25.39)	
Adequate	157 (36.18)	268 (41.74)	
Excessive	94 (21.66)	211 (32.87)	
Gestational age (wk), (median, IQR)	38.6 (38.1–39.2)	39.1 (38.2–39.5)	**<**.01
ART (%)	94 (21.66)	142 (22.12)	.86
Gravidity ≥ 3 (%)	110 (25.35)	133 (20.72)	.07

ART = assisted reproductive technology, BMI = body mass index, GDM = gestational diabetes mellitus, GWG = gestational weight gain, IQR = interquartile range.

Polyhydramnios was more frequently observed in the advanced primiparous women with GDM (5.76% vs 2.65%, *P* = .01). A significantly higher incidence rate of adverse pregnancy outcomes was found in advanced primiparous women with GDM, such as premature birth, low-birth weight, macrosomia, and NICU admission (all *P* < .05). Detailed information about the comparison of pregnancy outcomes was shown in Table 2.

**Table 2:** Comparison of pregnancy outcomes of advanced primiparous women with/without GDM.

Variables	GDM group (*n* = 434)	Non-GDM group (*n* = 642)	*P* value
PROM (%)	60 (13.82)	97 (15.11)	.56
Polyhydramnios (%)	25 (5.76)	17 (2.65)	.01
Hypertensive disorders of pregnancy (%)	72 (16.59)	90 (14.02)	.22
Hypothyroidism	53 (12.21)	72 (11.21)	.62
Cesarean delivery (%)	335 (77.19)	463 (72.12)	.06
Postpartum hemorrhage (%)	14 (3.22)	22 (3.43)	.86
Fetal distress (%)	53 (12.21)	64 (9.97)	.25
Premature birth (%)	51 (11.75)	51 (7.94)	.04
Low-birth weight (%)	42 (9.68)	37 (5.76)	.02
Macrosomia (%)	28 (6.45)	22 (3.43)	.02
NICU admission (%)	25 (5.76)	18 (2.80)	.02

GDM = gestational diabetes mellitus, NICU = neonatal intensive care unit, PROM = premature rupture of membrane.

The multivariate logistic regression models were built. After adjustment for baseline characteristics of maternal age, height, weight, prepregnancy BMI, GWG, ART, and gravidity, the ORs for the risk of adverse pregnancy outcomes according to GDM were presented in Table 3. GDM had almost twice the risk for developing polyhydramnios (adjusted OR: 1.94, 95% CI: 1.01–3.72, *P* = .04).

**Table 3:** The results for the odds ratios for the risk of adverse pregnancy outcomes according to GDM, after adjustment for baseline characteristics.

	OR	95% CI	*P* value
PROM	0.93	0.65–1.32	.67
Polyhydramnios	1.94	1.01–3.72	.04
Hypertensive disorders of pregnancy	0.99	0.69–1.40	.94
Hypothyroidism	1.08	0.73–1.59	.71
Cesarean delivery	1.03	0.76–1.39	.87
Postpartum hemorrhage	1.07	0.53–2.15	.85
Fetal distress	1.30	0.82–1.92	.20
Premature birth	1.31	0.86–2.01	.21
Low-birth weight	1.49	0.92–2.40	.10
Macrosomia	1.04	0.57–1.88	.91
NICU admission	1.73	0.91–3.31	.10

The risk for adverse pregnancy outcomes was analyzed using the odds ratio (OR) and 95% confidence interval (CI) by multivariate logistic regression models. Adjustment was made for baseline characteristics of maternal age, height, weight, prepregnancy body mass index, gestational weight gain, assisted reproductive technology, and gravidity.

CI = confidence interval, GDM = gestational diabetes mellitus, NICU = neonatal intensive care unit, OR = odds ratio, PROM = premature rupture of membrane.

Subgroup analysis was performed to explore the effect of glycaemic control on pregnancy outcomes (Table 4). Of the 434 advanced primiparous women with GDM, 381 had good glycaemic control, and 53 had poor glycaemic control. The poor glycaemic group showed a significantly higher incidence rate of polyhydramnios, hypertensive disorders of pregnancy, cesarean delivery, premature birth, low-birth weight, macrosomia, and NICU admission than the good glycaemic control group (all *P* < .05).

**Table 4:** Subgroup analysis was used to analyze the effect of glycaemic control of gestational diabetes mellitus on pregnancy outcomes in advanced primiparous women.

Variables	Good glycaemic control group (*n* = 381)	Poor glycaemic control group (*n* = 53)	*P* value
PROM (%)	52 (13.65)	8 (15.09)	.76
Polyhydramnios (%)	13 (3.41)	12 (22.64)	<.01
Hypertensive disorders of pregnancy (%)	55 (14.44)	17 (32.08)	<.01
Hypothyroidism (%)	45 (11.81)	8 (15.09)	.49
Cesarean delivery (%)	288 (75.59)	47 (88.68)	.03
Postpartum hemorrhage (%)	13 (3.41)	1 (1.89)	.32
Fetal distress (%)	47 (12.34)	6 (11.32)	.83
Premature birth (%)	19 (4.99)	32 (60.38)	<.01
Low-birth weight (%)	22 (5.77)	20 (37.74)	<.01
Macrosomia (%)	11 (2.89)	17 (32.08)	<.01
NICU admission (%)	13 (3.41)	12 (22.64)	<.01

GDM = gestational diabetes mellitus, NICU = neonatal intensive care unit, PROM = premature rupture of membrane.

## 4. Discussion

Socio-economic development and assisted reproductive technologies have led to delays in childbearing. Age is an inherent risk factor in the development of metabolic diseases.^[13]^ With the widespread use of glucose diagnostic monitoring technology and pregnancy checkups, the detection rate of gestational diabetes has increased to a certain degree. Many studies reported AMA and GDM were related to adverse pregnancy outcomes.^[6,14]^ However, no research has addressed the association of GDM with pregnancy outcomes in advanced primiparous women. Thus, we investigated GDM and pregnancy outcomes in advanced primiparous women in the current study. Our findings demonstrated that GDM was an independent risk of developing polyhydramnios in advanced primiparous women. At the same time, good glycaemic control in diabetics advanced primiparous women could reduce adverse pregnancy outcomes.

Studies showed that the risk of developing GDM is higher in older pregnant women, and AMA (≥35 years) was a well-documented and unmodifiable risk factor for GDM.^[15]^ Obesity and inadequate GWG were other known risk factors for GDM.^[16,17]^ The current study included 1076 advanced primiparous women and found the prepregnancy weight and BMI in the GDM group were much higher than those in the non-GDM group; due to metabolic changes and inactive lifestyle during pregnancy, especially the decrease in insulin sensitivity in late pregnancy, suggesting obese pregnant women with a higher risk of dyshomeostasis during pregnancy. The current study found the GWG was much lower in the GDM group than in the GDM group (median, IQR: 11.50 [8.0–15.0] vs 14.0 [11.0–17.0], *P* < .01). Similarly, a study from Spain also reported that GWG was lower in diabetic women (10.88 ± 6.46 vs 12.30 ± 5.42 kg; *P* = .013).^[18]^ The current study also reported a larger proportion of advanced primiparous women with GDM had inadequate GWG (42.17%).

As is well-known, GDM is related to adverse pregnancy outcomes, such as hypertriglyceridemia, PROM, premature birth, postpartum hemorrhage, low-birth weight, macrosomia, fetal distress, etc.^[19]^ The incidences of adverse pregnancy outcomes between the GDM group and non-GDM group in advanced primiparous women were also compared. The current study found the incidence of polyhydramnios, premature birth, low-birth weight, macrosomia, and NICU admission was higher in the GDM group (all *P* < .05). Subsequently, a multiple logistic regression model was conducted to control potential confounders for adverse pregnancy outcomes based on GDM. Our results showed that GDM was an independent risk of developing polyhydramnios (adjusted OR = 1.94, 95% CI: 1.01–3.72). A prospective cohort study of 694 pregnant women in Ethiopia found a higher incidence of gestational hypertension, PROM, prenatal hemorrhage, and postpartum hemorrhage in pregnant women with GDM.^[20]^ Polyhydramnios can result from a multifactorial etiology. Many fetal and maternal conditions, such as glycemic impairments, could lead to amniotic fluid abnormalities during pregnancy.^[21]^ Excess glucose entered the fetus through the placenta when maternal blood glucose increased. The high levels of sugar entering the fetus through the placenta promoted hyperglycemia and hyperosmolar diuresis, which led to increased urinary excretion, excess maternal amniotic fluid, and the incidence of PROM and premature birth.^[22]^ In contrast, this study found no significant difference in the incidence of PROM among the GDM group and non-GDM group. It may be explained by the cesarean section chosen as the preferred mode of delivery by more and more advanced primiparous women, avoiding the occurrence of PROM. Although polyhydramnios was associated with diabetes during pregnancy,^[23]^ the association with the timing of the onset of GDM was not sure. More studies are needed to explore the potential effect of GDM on polyhydramnios.

To observe the effects of glycaemic control of GDM on pregnancy outcomes in advanced primiparous women, a subgroup analysis among the 434 women with GDM was conducted. There were 381 women with good glycaemic control and 53 women with poor glycaemic control. As expected, good glycaemic control reduced adverse pregnancy outcomes, such as polyhydramnios, hypertensive disorders of pregnancy, cesarean delivery, premature birth, low-birth weight, macrosomia, and NICU admission. Our study also highlighted the importance of blood glucose in primiparous women of advanced age. Lifestyle behavior change is essential to managing GDM for many women.^[24]^ Lifestyle behavior change mainly includes dietary and exercise intervention. Although there is no consensus on which nutritional approach should be adopted for GDM, increasing evidence has reported that a low glycemic index diet was beneficial for diabetes in the past decade.^[25]^ A meta-analysis by Li et al showed that exercise prescription for GDM patients could manage GDM and improve adverse pregnancy outcomes effectively.^[26]^ Pregnant women with GDM are recommended to exercise at least twice a week for at least 20 to 50 minutes with at least moderate intensity.^[27]^

However, there are some limitations to be issued in this study. Considering it was a retrospective study in a single center, there may be unavoidable information bias and selection bias. The current study lacked adjustment for individualized maternal glycaemic control, which may also affect maternal and neonatal outcomes. Therefore, more studies are needed to examine the impact of glycaemic control on maternal and neonatal outcomes in advanced primiparous women. Medical institutions should take into account their management capabilities and the results of OGTT examinations and glucose monitoring in the later stages of gestational diabetes to stratify pregnant women, especially those with more severe glucose metabolism abnormalities, to improve their compliance and self-management capabilities to reduce the occurrence of adverse pregnancy outcomes and promote maternal and infant health.

## 5. Conclusion

Our findings supplemented the limited evidence of GDM in advanced primiparous women, providing valuable support for GDM Management in maternal women with AMA. GDM was an independent risk factor for developing polyhydramnios in diabetics advanced primiparous women. Good glycaemic control in diabetics advanced primiparous women could effectively reduce adverse pregnancy outcomes. It is necessary to identify high-risk individuals early and take preventive and intervention measures to reduce the risk of GDM and adverse pregnancy outcomes.

## Author contributions

**Data curation:** Hong Yang, Chanyun Xiao, Jiahui Tu.

**Formal analysis:** Hong Yang, Chanyun Xiao.

**Validation:** Hong Yang, Chanyun Xiao, Jiahui Tu.

**Visualization:** Hong Yang, Chanyun Xiao, Jiahui Tu.

**Writing—original draft:** Hong Yang, Chanyun Xiao, Jiahui Tu.

**Writing—review & editing:** Hong Yang, Chanyun Xiao, Jiahui Tu.

**Conceptualization:** Hong Yang, Jiahui Tu.

**Project administration:** Jiahui Tu.

**Supervision:** Jiahui Tu.

## Supplementary Material





## References

[1] Glick I Kadish E Rottenstreich M. Management of pregnancy in women of advanced maternal age: improving outcomes for mother and baby. Int J Womens Health. 2021;13:751–9. 34408501 10.2147/IJWH.S283216 PMC8364335

[2] Attali E Yogev Y. The impact of advanced maternal age on pregnancy outcome. Best Pract Res Clin Obstet Gynaecol. 2021;70:2–9. 32773291 10.1016/j.bpobgyn.2020.06.006

[3] McIntyre HD Catalano P Zhang C. Gestational diabetes mellitus. Nat Rev Dis Primers. 2019;5:47. 31296866 10.1038/s41572-019-0098-8

[4] Vounzoulaki E Khunti K Abner SC. Progression to type 2 diabetes in women with a known history of gestational diabetes: systematic review and meta-analysis. BMJ. 2020;369:m1361. 32404325 10.1136/bmj.m1361 PMC7218708

[5] He XL Hu XJ Luo BY. The effects of gestational diabetes mellitus with maternal age between 35 and 40 years on the metabolite profiles of plasma and urine. BMC Pregnancy Childbirth. 2022;22:174. 35236326 10.1186/s12884-022-04416-5 PMC8892719

[6] Ye W Luo C Huang J. Gestational diabetes mellitus and adverse pregnancy outcomes: systematic review and meta-analysis. BMJ. 2022;377:e067946. 35613728 10.1136/bmj-2021-067946 PMC9131781

[7] Zheng QX Wang HW Jiang XM. Prepregnancy body mass index and gestational weight gain are associated with maternal and infant adverse outcomes in Chinese women with gestational diabetes. Sci Rep. 2022;12:2749. 35177745 10.1038/s41598-022-06733-3 PMC8854692

[8] Xu HB Li MH Tang XF. The relationship between poor glycaemic control at different time points of gestational diabetes mellitus and pregnancy outcomes. J Obstet Gynaecol. 2022;42:2979–86. 36149633 10.1080/01443615.2022.2124852

[9] Deng L Ning B Yang H. Association between gestational diabetes mellitus and adverse obstetric outcomes among women with advanced maternal age: a retrospective cohort study. Medicine (Baltimore). 2022;101:e30588. 36221369 10.1097/MD.0000000000030588 PMC9542683

[10] Vince K Perković P Matijević R. What is known and what remains unresolved regarding gestational diabetes mellitus (GDM). J Perinat Med. 2020;48:757–63. 32827397 10.1515/jpm-2020-0254

[11] Chen YH Fu L Hao JH. Influent factors of gestational vitamin D deficiency and its relation to an increased risk of preterm delivery in Chinese population. Sci Rep. 2018;8:3608. 29483547 10.1038/s41598-018-21944-3 PMC5827025

[12] Institute of M, National Research Council Committee to Reexamine IOMPWG. The National Academies Collection: Reports funded by National Institutes of Health [Copyright © 2009, National Academy of Sciences]. In: Rasmussen KM Yaktine AL, eds. Weight Gain During Pregnancy: Reexamining the Guidelines. Washington, DC: National Academies Press (US); 2009. 20669500

[13] Cao Q Zheng R He R. Age-specific prevalence, subtypes and risk factors of metabolic diseases in Chinese adults and the different patterns from other racial/ethnic populations. BMC Public Health. 2022;22:2078. 36376828 10.1186/s12889-022-14555-1 PMC9664823

[14] Guarga Montori M Álvarez Martínez A Luna Álvarez C. Advanced maternal age and adverse pregnancy outcomes: a cohort study. Taiwanese J Obstet Gynecol. 2021;60:119–24. 33494983 10.1016/j.tjog.2020.11.018

[15] Li Y Ren X He L. Maternal age and the risk of gestational diabetes mellitus: a systematic review and meta-analysis of over 120 million participants. Diabetes Res Clin Pract. 2020;162:108044. 32017960 10.1016/j.diabres.2020.108044

[16] Yong HY Mohd Shariff Z Mohd Yusof BN. Independent and combined effects of age, body mass index and gestational weight gain on the risk of gestational diabetes mellitus. Sci Rep. 2020;10:8486. 32444832 10.1038/s41598-020-65251-2 PMC7244566

[17] Sun Y Shen Z Zhan Y. Effects of pre-pregnancy body mass index and gestational weight gain on maternal and infant complications. BMC Pregnancy Childbirth. 2020;20:390. 32631269 10.1186/s12884-020-03071-y PMC7336408

[18] de la Torre NG Assaf-Balut C Jiménez Varas I. Effectiveness of following mediterranean diet recommendations in the real world in the incidence of gestational diabetes mellitus (GDM) and adverse maternal-foetal outcomes: a prospective, universal, interventional study with a single group. The St Carlos study. Nutrients. 2019;11:1210. 31141972 10.3390/nu11061210 PMC6627921

[19] Lende M Rijhsinghani A. Gestational diabetes: overview with emphasis on medical management. Int J Environ Res Public Health. 2020;17:9573. 33371325 10.3390/ijerph17249573 PMC7767324

[20] Muche AA Olayemi OO Gete YK. Effects of gestational diabetes mellitus on risk of adverse maternal outcomes: a prospective cohort study in Northwest Ethiopia. BMC Pregnancy Childbirth. 2020;20:73. 32013909 10.1186/s12884-020-2759-8 PMC6998275

[21] Parveen N Hassan SU Zahra A. Early-onset of gestational diabetes vs. late-onset: can we revamp pregnancy outcomes? Iran J Public Health. 2022;51:1030–9. 36407740 10.18502/ijph.v51i5.9418 PMC9643226

[22] Aviram A Guy L Ashwal E. Pregnancy outcome in pregnancies complicated with gestational diabetes mellitus and late preterm birth. Diabetes Res Clin Pract. 2016;113:198–203. 26810272 10.1016/j.diabres.2015.12.018

[23] Li J Yan J Ma L. Effect of gestational diabetes mellitus on pregnancy outcomes among younger and older women and its additive interaction with advanced maternal age. Front Endocrinol. 2023;14:1158969. 10.3389/fendo.2023.1158969 PMC10206299 37234802

[24] American Diabetes Association. 14. Management of diabetes in pregnancy: standards of medical care in diabetes—2021. Diabetes Care. 2021;44(Suppl 1):S200–S210. 33298425 10.2337/dc21-S014

[25] Filardi T Panimolle F Crescioli C. Gestational diabetes mellitus: the impact of carbohydrate quality in diet. Nutrients. 2019;11:1549. 31323991 10.3390/nu11071549 PMC6683084

[26] Li X Luo R Qiao B. Exercise intervention improves blood glucose levels and adverse pregnancy outcomes in GDM Patients: a meta-analysis. Comput Math Methods Med. 2022;2022:9287737. 36238491 10.1155/2022/9287737 PMC9553359

[27] Laredo-Aguilera JA Gallardo-Bravo M Rabanales-Sotos JA. Physical activity programs during pregnancy are effective for the control of gestational diabetes mellitus. Int J Environ Res Public Health. 2020;17:6151. 32847106 10.3390/ijerph17176151 PMC7503359

