# Supplementary material for: The effect of gestational diabetes mellitus on pregnancy outcomes in advanced primiparous women: A retrospective study
Source: Medicine (Baltimore). 2024 Mar 29;103(13):e37570. doi: 10.1097/MD.0000000000037570 (PMC10977535; doi:10.1097/MD.0000000000037570)
Supplement: Supplementary file 1 [file medi-103-e37570-s001.docx]

**Supplementary Table 1. Pre-pregnancy BMI categorization according to the WHO cut-points for Asian adults.**

|  | Underweight | Normal weight | Overweight | Obese |
| --- | --- | --- | --- | --- |
| Pre-pregnancy BMI in kg/m^2^ | < 18.5 | 18.5–24.9 | 25–29.9 | ≥ 30 |

BMI: body mass index.
