# Supplementary material for: The effect of gestational diabetes mellitus on pregnancy outcomes in advanced primiparous women: A retrospective study
Source: Medicine (Baltimore). 2024 Mar 29;103(13):e37570. doi: 10.1097/MD.0000000000037570 (PMC10977535; doi:10.1097/MD.0000000000037570)
Supplement: Supplementary file 2 [file medi-103-e37570-s002.docx]

**Supplementary Table 2. Categorization according to the 2009 Institute of Medicine (IOM) recommendations.**

|  | Underweight | Normal weight | Overweight | Obese |
| --- | --- | --- | --- | --- |
| Inadequate | GWG < 12.5 kg | < 11.5 kg | < 7 kg | < 5 kg |
| Adequate | 12.5 ≤ GWG ≤ 18 kg | 11.5 ≤ GWG ≤ 16 kg | 7 ≤ GWG ≤ 11.5 kg | 5 ≤ GWG ≤ 9 kg |
| Excessive | GWG > 18 kg | >16 kg | > 11.5 kg | > 9 kg |

GWG: gestational weight gain.
